# Nosocomial Outbreak of OXA-48-Producing *Klebsiella pneumoniae* in a Chinese Hospital: Clonal Transmission of ST147 and ST383

**DOI:** 10.1371/journal.pone.0160754

**Published:** 2016-08-04

**Authors:** Ling Guo, Jingna An, Yanning Ma, Liyan Ye, Yanping Luo, Chuanmin Tao, Jiyong Yang

**Affiliations:** 1 Department of Microbiology, Chinese PLA General Hospital, Beijing, China; 2 Department of Laboratory Medicine, West China Hospital, Sichuan University, Chengdu, China; Universite Clermont Auvergne, FRANCE

## Abstract

**Background:**

In China, the spread and outbreak of OXA-48-producing *Enterobacteriaceae* remains largely unknown.

**Methods:**

OXA-48-producing isolates were analyzed for genetic relatedness by pulsed-field gel electrophoresis (PFGE), antimicrobial susceptibility by E-test, and sequence type (ST) by multilocus sequence typing. S1-PFGE and southern blotting were used for plasmid profiling, and PCR and subsequent sequencing were performed to determine the genetic environment of *bla*_OXA-48_ gene.

**Results:**

In total, 37 non-duplicated OXA-48-producing *K*. *pneumoniae* (OXAKp) isolates were recovered. From December 2013 to August 2014, an outbreak was observed at a respiratory ICU. The 37 isolates of *K*. *pneumoniae* were categorized into four PFGE types (A, B, C, and D). The predominant strains associated with the outbreak were strains with PFGE type A and B, which belonged to ST383 and ST147, respectively. Plasmid sequencing revealed that the *bla*_OXA-48_-carrying plasmid is 69,069 bp in length and belongs to the IncL/M incompatibility group. Sequence analysis revealed that the IS*1999* element was located upstream of the *bla*_OXA-48_ gene and was truncated by IS*1R*.

**Conclusions:**

In this study, the dissemination and outbreak of OXAKp isolates were clonal, and ST147 and ST383 *K*. *pneumoniae* were the predominant clones that were associated with the outbreak. Meanwhile, the horizontal transfer of plasmids potentially mediate the spread of *bla*_OXA-48_ gene between different *K*. *pneumoniae* strains.

## Introduction

Global spread of carbapenemase-producing *Klebsiella pneumoniae* is a growing clinical problem and public health threat [1]. In 2004, a novel class D carbapenemase, OXA-48 oxacillinase, was identified in a clinical *K*. *pneumoniae* isolate [2]. Since then, OXA-48-producing *K*. *pneumoniae* (OXAKp) has been primarily reported in Turkey, and in countries of the Middle East, North Africa, and Europe [1,3]. In some countries, OXAKp accounted for the majority of carbapenemase-producing *Enterobacteriaceae* [4,5].

To date, OXA-48 and its several variants have been identified in *Enterobacteriaceae* [6,7]. These variants differ from OXA-48 by one to five amino acid substitutions [6]. OXA-48-type carbapenemases weakly hydrolyze carbapenems, but does not exhibit activity against extended-spectrum cephalosporins and aztreonam. However, some OXA-48-like variants confer resistance to broad spectrum cephalosporins and can lead to high levels of carbapenems and cephalosporins resistance that is associated with impaired permeability or the production of extended-spectrum β-lactamases (ESBLs) [3]. A recent study demonstrated that the OXA-48-like variants possessed different carbapenems hydrolytic properties, while OXA-163 variant did not exhibit significant carbapenemase activity [8].

In Europe, diverse sequence types (STs) of dominant OXAKp have been identified in outbreaks or solitary case reports (STs 11, 14, 15, 16, 17, 45, 101, 104, 147, 326, 392, 395 and 405) [9,10,11,12,13,14]. In Asia, OXAKp ST15 have been identified in India [13], and OXAKp ST11 and ST116 were present in Taiwan [15].

The *bla*_OXA-48_ gene is found on a IncL/M-type self-transferable plasmid of approximately 62 kb that was disseminated in various enterobacterial species [3]. Other types of *bla*_OXA-48_-carrying plasmids (e.g. IncA/C, IncFIA, and IncF) have also been identified in *Enterobacteriaceae* [11,12,15]. The *bla*_OXA-48_ gene is flanked by two IS*1999* elements to form a functional composite transposon Tn*1999*, which does not carry any other antibiotic resistance gene [2,16]. In addition, a novel Tn*1999* transposon variant (Tn*1999*.*2*) has been identified in which the IS*1999* element was located upstream of the *bla*_OXA-48_ gene and truncated by IS*1R* [17]. Two other Tn*1999* transposon derivatives (Tn*1999*.*3* and Tn*1999*.*4*) were also discovered located downstream of the *bla*_OXA-48_ gene and truncated by IS*1R* or by a more complicated genetic structure (named Tn*2015*) [18,19].

Currently, OXA-48-producing *Enterobacteriaceae* has been reported only in regions of Taiwan [15] and has not been found in other regions of China. In this study, we report a nosocomial outbreak of OXAKp at our hospital involving 34 patients. The phenotypic and genotypic characteristics of OXAKp isolates were analyzed.

## Materials and Methods

### Bacterial isolates

All clinical enterobacterial isolates were collected from a 4000-bed tertiary-care hospital and were identified by VITEK^®^ MS (bioMérieux SA, Marcy-l'Etoile, France). No ethical approval was obtained for using the clinical samples since they were collected during routine bacteriologic analyses in public hospitals. All data were anonymously analyzed.

### Antimicrobial susceptibility testing

The MICs of cefotaxime (CTX), piperacillin-tazobactam (TZP), imipenem (IMP), meropenem (MEM), ertapenem (ETP), amikacin (AK) and levofloxacin (LEV) were measured by E-test (AB bioMérieux, Solna, Sweden). *E*. *coli* ATCC 25922 was used as the quality control strains for antimicrobial susceptibility testing. Results were interpreted according to the interpretive standards of the Clinical Laboratory Standards Institute [20].

### Detection of specific porin and resistance genes

The isolates that exhibited non-susceptibility to carbapenems were screened for *bla*_OXA-48_ by PCR amplification and subsequent amplicon sequencing as previously described [2]. PCR detection of *bla*_CTX-M_ genes was performed as previously described [21]. The DNA sequences of outer membrane protein (OMP) genes were analyzed as previously described [22]. All PCR products were purified and sequenced.

### Pulsed-field gel electrophoresis (PFGE) and MLST analysis

PFGE with *Xba*I was performed for OXAKp isolates [23]. *Salmonella* ser. Braenderup strain (H9812) was used as a reference standard for PFGE. MLST was carried out according to protocols provided on MLST websites (http://www.pasteur.fr/recherche/genopole/PF8/mlst/Kpneumoniae).

### Plasmid analysis and Southern blot

The transferability of plasmids was demonstrated by conjugation experiments [23]. Sodium azide resistant *E*. *coli* J53 was used as the recipient for conjugation testing. The resistance plasmids were typed by using several simplex and multiplex PCR [24,25]. A *bla*_OXA-48_ probe was generated by labeling a *bla*_OXA-48_ PCR product by the PCR DIG Probe Synthesis Kit (Roche Applied Sciences, Mannheim, Germany). Plasmid analysis was performed in one representative of each PFGE profile, and the S1-PFGE and Southern blot were performed [23]. A *bla*_OXA-48_-carrying plasmid from type A strain was sequenced using the Illumina MiSeq system [26].

### Genetic environment analysis of *bla*_OXA-48_ gene

To confirm the upstream genetic structures of the *bla*_OXA-48_ gene, a primer pair Tn1999-F (5’-AGTTCTGGGCAGTATTGGTGT) and Tn1999-R (5’-ACACGCATAACGTCCCCTTG) were used to amplify a 192 bp or 970 bp fragments from the IS*1999* (GenBank no. JN626286) or the IS*1R*-truncated IS*1999* (GenBank no. JN714122), respectively. All PCR products were purified and sequenced.

## Results

### Emergence and outbreak of OXAKp

**Fig 1:**
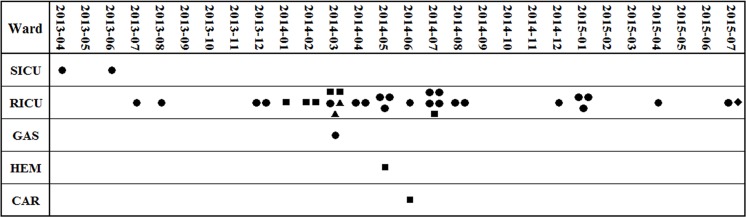
The date, frequency and location of emergence of OXAKp strains with different PFGE types. The horizontal coordinate shows the date on which OXAKp strains were recovered. The wards were listed in the longitudinal coordinate. SICU: surgical ICU; RICU: respiratory ICU; GAS: gastroenterology department; HEM: hematology department; CAR: cardiovascular department. Clinical isolates with different PFGE types are indicated by various symbols: ●: type A;■: type B; **▲**: type C; ◆: type D.

In total, 2310 *K*. *pneumoniae* isolates were recovered from various clinical specimens at our hospital between March 2013 and July 2015, and 247 (10.69%) of these were found to be non-susceptible to carbapenems. During this period, 37 non-duplicated OXAKp isolates were recovered from 34 patients in two ICUs and three other clinical wards (Fig 1). There were three patients from whom the OXAKp isolates were recovered from different sites of the same patient. Among these isolates, 23 (62.2%) were recovered from sputum, while 5 and 9 strains were recovered from blood and urine sample, respectively. In April 2013, OXAKp first appeared at the surgical ICU. This followed by an outbreak from December 2013 to August 2014 at a respiratory ICU (RICU) (Fig 1). Among 34 patients with OXAKp isolates, 22 (64.7%) died. Nine patients (26.5%) showed improvement, while two patients and another patient were successfully cured and discharged, respectively.

### PFGE and MLST analysis

The 37 isolates of *K*. *pneumoniae* were categorized into four PFGE types (A, B, C, and D). The majority of the isolates belonged to types A and B, while types C and D isolates were detected in two and one samples respectively (Table 1). Before December 2013, only type A strain was sporadically found at SICU and RICU. Eight months after the emergence of type A OXAKp strain, only sporadic cases were identified in two ICUs. This followed by an outbreak in a respiratory ICU in the next nine months. Meanwhile, new OXAKp clones (types B and C) emerged and were found spread to other wards (Fig 1). MLST was performed in one representative of each PFGE profile and three sequence types (STs) were identified. Types A and D strains belonged to ST383, while types B and C were categorized with ST147 and ST13, respectively.

**Table 1:** Phenotypic and genotypic characteristics of OXA-48-producing *K*. *pneumoniae* isolates.

No.	Isolate No.	Source	PFGE type	ST	*ompk35*	*ompk36*	CTX-M	Minimal inhibitory concentration (mg/L)						
								CTX	TZP	IPM	MEM	ETP	AK	LEV
1	IR5065	Blood	A	ST383	Truncated*	Intact	14	>256	>256	2	2	32	64	>32
2	IR5067	Urine	A	ST383	Truncated*	Intact	14	>256	>256	4	1	4	>256	>32
3	IR5070	Sputum	A	ST383	Truncated*	Intact	14	>256	>256	2	1	4	128	>32
4	IR5075	Blood	A	ST383	Truncated*	Intact	14	>256	>256	>32	>32	>32	64	>32
5	IR5082	Blood	A	ST383	Truncated*	Intact	14	>256	>256	2	1	32	128	>32
6	IR5083	Sputum	A	ST383	Truncated*	Intact	14	>256	>256	>32	>32	>32	>256	>32
7	IR5088	Sputum	A	ST383	Truncated*	Intact	14	>256	>256	>32	>32	>32	>256	>32
8	IR5090	Urine	A	ST383	Truncated*	Intact	14	>256	>256	8	>32	>32	>256	>32
9	IR5097	Urine	A	ST383	Truncated*	Intact	14	>256	>256	>32	>32	>32	>256	>32
10	IR5098	Sputum	A	ST383	Truncated*	Intact	14	>256	>256	>32	24	>32	96	>32
11	IR5099	Sputum	A	ST383	Truncated*	Intact	14	>256	>256	>32	>32	>32	128	>32
12	IR5100	Sputum	A	ST383	Truncated*	Intact	14	>256	>256	2	1	32	48	>32
13	IR5602	Sputum	A	ST383	Truncated*	Intact	14	>256	>256	8	>32	>32	48	>32
14	IR5604	Sputum	A	ST383	Truncated*	Intact	14	>256	>256	>32	>32	>32	>256	>32
15	IR5607	Urine	A	ST383	Truncated*	Intact	14	>256	>256	4	2	4	64	>32
16	IR5609	Urine	A	ST383	Truncated*	Intact	14	>256	>256	>32	>32	>32	128	>32
17	IR5610	Sputum	A	ST383	Truncated*	Intact	14	>256	>256	2	1	4	64	>32
18	IR5614	Urine	A	ST383	Truncated*	Intact	14	>256	>256	2	1	4	96	>32
19	IR5615	Sputum	A	ST383	Truncated*	Intact	14	>256	>256	4	4	32	128	>32
20	IR5618	Sputum	A	ST383	Truncated*	Intact	14	>256	>256	>32	>32	>32	>256	>32
21	IR5629	Urine	A	ST383	Truncated*	Intact	14	>256	>256	2	4	4	>256	>32
22	IR5630	Sputum	A	ST383	Truncated*	Intact	14	>256	>256	2	4	4	32	>32
23	IR5632	Blood	A	ST383	Truncated*	Intact	14	>256	>256	>32	>32	>32	>256	>32
24	IR5633	Sputum	A	ST383	Truncated*	Intact	14	>256	>256	2	4	4	64	>32
25	IR5639	Sputum	A	ST383	Truncated*	Intact	14	>256	>256	>32	>32	>32	64	>32
26	IR5647	Sputum	A	ST383	Truncated*	Intact	14	>256	>256	2	4	4	64	>32
27	IR5085	Sputum	B	ST147	Intact	Intact	14, 15	>256	>256	4	2	16	>256	>32
28	IR5086	Sputum	B	ST147	Intact	Intact	14, 15	>256	>256	4	1	16	64	>32
29	IR5087	Sputum	B	ST147	Intact	Intact	14, 15	>256	>256	4	1	8	64	>32
30	IR5089	Urine	B	ST147	Intact	Intact	14, 15	>256	>256	2	1	2	64	>32
31	IR5092	Sputum	B	ST147	Intact	Intact	14, 15	>256	>256	4	1	4	64	>32
32	IR5601	Blood	B	ST147	Intact	Intact	14, 15	>256	>256	2	1	8	64	>32
33	IR5603	Sputum	B	ST147	Intact	Intact	14, 15	>256	>256	4	1	6	>256	>32
34	IR5612	Sputum	B	ST147	Intact	Intact	14, 15	>256	>256	2	1	4	64	>32
35	IR5093	Urine	C	ST13	Intact	Intact	14, 15	>256	>256	2	1	16	>256	>32
36	IR5094	Sputum	C	ST13	Intact	Intact	14, 15	>256	>256	4	4	16	64	>32
37	IR5649	Sputum	D	ST383	Truncated*	Intact	14	>256	>256	8	4	16	64	>32

**CTX:** cefotaxime, **TZP:** piperacillin-tazobactam, **IMP:** imipenem, **MEM:** meropenem, **ETP:** ertapenem, **AK:** amikacin.

*: truncated by IS*1R*.

### Antimicrobial susceptibilities

The antimicrobial susceptibility patterns for the isolates are listed in Table 1. All *K*. *pneumoniae* isolates presented resistance to CTX and TZP and exhibited heterogeneous carbapenem resistance patterns.

### OMP and CTX-M beta-lactamase genes

**Fig 2:**
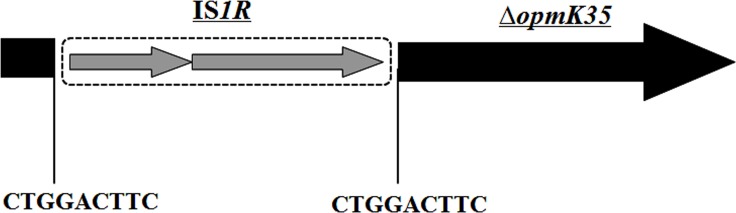
Schematic representation IS*1R*-truncated *opmK35* gene. Open reading frames are shown as horizontal boxes with an arrow indicating the orientation of the coding sequence. Duplications of the target site are represented by the vertical line.

No mutations were found in the *ompK36* coding region. For the *ompk35* gene, an intact open reading frame of 738 bp was found among types B and C isolates, whereas a much larger DNA fragments (1505 bp) were amplified from types A and D isolates. Sequencing analysis found that an additional insertion sequence, IS*1R* (768 bp), was inserted after nucleotide position 105 of the *ompK35* gene. Furthermore, a 9 bp duplication of the target site (CTGGACTTC) was identified (Fig 2). Types B and C isolates produced both CTX-M-14 and CTX-M-15, while only CTX-M-14 was detected among types A and D isolates (Table 1).

### Plasmid analysis and genetic environment of *bla*_OXA-48_ gene

S1-PFGE and southern blot analysis showed that the *bla*_OXA-48_ gene in all types of strains was located on an approximately 60 kb plasmid. Plasmid sequencing revealed that the *bla*_OXA-48_-carrying plasmid is 69,069 bp in length and belongs to the IncL/M incompatibility group. A BLAST search against all completely sequenced *bla*_OXA-48_-harboring plasmids in GenBank (http://www.ncbi.nlm.nih.gov/GenBank/) showed that the plasmid analyzed in this study displayed overall nucleotide identity (99.28%) to pOXA48-PM (GenBank: KP025948.1), a *bla*_OXA-48_-carrying IncL/M plasmid from a *Proteus mirabilis* strain (Pm-OXA-48) [27]. Sequence analysis revealed that the IS*1999* element was located upstream of the *bla*_OXA-48_ gene and was truncated by IS*1R*.

## Discussion

OXA-48 oxacillinase was identified in a *K*. *pneumoniae* isolate from Turkey in 2001 [2].

Since then, OXAKp isolates have been detected worldwide [3]. In Asia, OXAKp have only been identified in India [13] and Taiwan area of China [15]. In this study, 37 OXAKp isolates were collected between March 2013 and July 2015. A total of 247 clinical *K*. *pneumoniae* isolates exhibited non-susceptibility to carbapenems. In general, 14.98% (37/247) of clinical *K*. *pneumoniae* isolates that were non-susceptible to carbapenem produced OXA-48. PFGE analysis demonstrated that the majority (n = 26) of OXAKp isolates belonged to the same type (A clone) followed by type B clone (n = 8). OXAKp mainly disseminate in the respiratory ICU (Fig 1) and appeared to be clonal. Hospital outbreaks linked to patient transfer have also been observed in some European countries [3,4,5]. In France, OXAKp presents two-thirds of the carbapenemase-producing enterobacteriaceae [4]. Therefore, it is very important to recognize the impact of clonal dissemination on the prevalence of OXAKp and to strengthen the surveillance and reporting system of OXAKp in hospital infection control measures. In this study, 22 of 34 (64.7%) patients died, possibly from illnesses related to OXAKp-associated infections. However, most patients were hospitalized in the ICU and presented with severe conditions. As a result, it is difficult to determine whether the death of the patients were associated with the infections or adverse physical conditions. It has been confirmed that antimicrobial therapy is strongly associated with patient survival [28]. However, a high mortality rate was observed, although most patients had received carbapenems therapy in this study. Therefore, the mortality may be associated with the clinical conditions of the patients upon infection, rather than with infection itself.

OXAKp exhibits high-level carbapenem resistance when OXA-48 carbapenemases are associated with the production of ESBLs and impaired permeability [3]. In this study, type A and type B isolates produced both OXA-48 and CTX-M; however, none of these isolates exhibited high-levels of carbapenem resistance (Table 1). In addition, although all of the *ompk35* genes have been truncated by IS*1R*, about half of the type A isolates also presented low-level carbapenems resistance (Table 1). It is unclear to what extent the impacts of ESBLs and impaired outer membrane protein are on the carbapenem resistance of OXAKp. In most clinical microbiology laboratories in China, isolates with carbapenem-sensitive phenotype were undetected, regardless of whether they produce carbapenemases or not. The weak hydrolytic ability of OXA-48 to carbapenems may lead to an underestimation of the prevalence of OXA-48-producing isolates in China, and calls for changes in clinical microbiology analysis procedures that would enhance phenotypic and molecular detection of OXA-48 types of carbapenems.

In outbreaks of OXAKp worldwide, diverse sequence types of OXAKp have been reported. This included several dominant ones (e.g., STs 11, 14, 15, 16, 17, 45, 101, 104, 147, 326, 392, 395 and 405) [9,10,11,12,13]. In this study, the predominant clone of OXAKp belonged to ST383 and ST147, which were found in 27 and 8 isolates, respectively. Outbreaks of OXAKp ST147 are common in Europe [9,10,11,29], whereas prevalence of OXAKp ST383 have been sporadically reported in the United Kingdom [29]. ST383 contributes significantly to the dissemination of the VIM-producing *K*. *pneumoniae* [30,31,32], suggesting that the carbapenem-resistant phenotype may confer some fitness advantage to the spread of the pathogen. To our knowledge, this is the first study to report the outbreak of OXAKp ST383 worldwide.

In this study, all of the strains carry plasmids of the same size and incompatibility type (approximately 70 kb, IncL/M), suggesting that horizontal transfer of *bla*_OXA-48_-carrying plasmid may have occurred during the outbreak. The high nucleotide sequence homology between the *bla*_OXA-48_-carrying plasmid analyzed in this study and the previously reported plasmid sequences (i.e., *bla*_OXA-48_-carrying IncL/M plasmid from a *Proteus mirabilis* strain [Pm-OXA-48] [27]) also reveals possible sources of the plasmid. Other studies have confirmed that a single IncL/M plasmid of approximately 62 kb is the main source of the *bla*_OXA-48_ gene disseminated in a variety of enterobacterial species [16], and that the inter-genus transfer of *bla*_OXA-48_-carrying plasmids might occur more frequently *in vivo* than previously estimated by *in vitro* experiments [33]. All of these data indicated the important role of the self-conjugative IncL/M plasmids with approximately 60–70 kb in size on the prevalence *bla*_OXA-48_ gene. The genetic environment of the *bla*_OXA-48_ gene has been characterized as a functional composite transposon, which was identified as Tn*1999* [2,16] and several isoforms (Tn*1999*.*2*, Tn*1999*.*3* and Tn*1999*.*4*) [17,18,19]. For strains analyzed in this study, the genetic environment of the *bla*_OXA-48_ are consistent with those of Tn*1999*.*2*, Tn*1999*.*3*, or Tn*1999*.*4*. In these strain, the IS*1999* element is located upstream of the *bla*_OXA-48_ gene and is truncated by IS*1R* [19]. However, PCR and subsequent sequencing analyses failed to differentiate variants such as Tn*1999*.*2*, Tn*1999*.*2* inverted, Tn*1999*.*3*, Tn*1999*.*4*, or other new variants. Additional overlapping PCRs and sequencing analyses are needed to reveal the downstream sequence of *bla*_OXA-48_ gene.

In conclusion, a clonal dissemination and outbreak of OXAKp has been observed. ST147 and ST383 *K*. *pneumoniae* were the predominant clones that were associated with the outbreak. Meanwhile, the horizontal transfer of *bla*_OXA-48_-carrying plasmids potentially mediate the spread of *bla*_OXA-48_ gene among different *K*. *pneumoniae* strains.
